# Shared metabolic shifts in endothelial cells in stroke and Alzheimer’s disease revealed by integrated analysis

**DOI:** 10.1038/s41597-023-02512-5

**Published:** 2023-09-29

**Authors:** Guangyu Guo, Liyuan Fan, Yingxue Yan, Yunhao Xu, Zhifen Deng, Miaomiao Tian, Yaoqi Geng, Zongping Xia, Yuming Xu

**Affiliations:** 1 Department of Neurology, The First Affiliated Hospital of Zhengzhou University, Zhengzhou, Henan China; 2 NHC Key Laboratory of Prevention and treatment of Cerebrovascular Diseases, Zhengzhou, China; 3 Clinical Systems Biology Laboratories, the First Affiliated Hospital of Zhengzhou University, Zhengzhou, China; 4 Academy of Medical Sciences of Zhengzhou University, Zhengzhou, China; 5 Department of Endocrinology, The First Affiliated Hospital of Zhengzhou University, Zhengzhou, China

**Keywords:** Neurodegenerative diseases, Data integration

## Abstract

Since metabolic dysregulation is a hallmark of both stroke and Alzheimer’s disease (AD), mining shared metabolic patterns in these diseases will help to identify their possible pathogenic mechanisms and potential intervention targets. However, a systematic integration analysis of the metabolic networks of the these diseases is still lacking. In this study, we integrated single-cell RNA sequencing datasets of ischemic stroke (IS), hemorrhagic stroke (HS) and AD models to construct metabolic flux profiles at the single-cell level. We discovered that the three disorders cause shared metabolic shifts in endothelial cells. These altered metabolic modules were mainly enriched in the transporter-related pathways and were predicted to potentially lead to a decrease in metabolites such as pyruvate and fumarate. We further found that Lef1, Elk3 and Fosl1 may be upstream transcriptional regulators causing metabolic shifts and may be possible targets for interventions that halt the course of neurodegeneration.

## Introduction

Hemorrhagic stroke (HS), which occurs when intracerebral arterioles break, and ischemic stroke (IS), which occurs when a major cerebral artery is blocked, are the two main types of stroke^1,2^. As a type of neurological disease, stroke is the second major cause of death and a key contributor to disability-adjusted life years (DALYs). Approximately 101.47 million stroke events occurred globally in 2019, 12.2 million of which were new cases^3^. Studies have confirmed neurodegeneration secondary to stroke, which can cause progressive neuronal loss, cognitive and motor dysfunction, and ultimately dementia^4–6^.

Alzheimer’s disease (AD) is a dementia that is characterized by age-related declines in cognition and higher functions^7^. More than 40 million people worldwide are afflicted by this neurodegenerative illness^8^. The main biochemical indicator of AD is the development of intracellular neurofibrillary tangles and extracellular deposits of the peptide beta amyloid as senile plaques in subcortical areas of the brain^9,10^. Recent studies have shown that metabolic disturbances, oxidative stress, and bioenergetic deficits are associated with AD and might contribute to its progression^11–13^.

A growing number of studies have suggested that stroke and AD may have some underpinnings in common^14,15^. There is substantial evidence demonstrating a significant overlap between vascular risk factors and the pathogenesis of AD, as well as impaired cognition^16–18^. These vascular risk factors include insulin resistance, diabetes, obesity, hyperhomocystinemia, and high cholesterol levels, all of which have been linked to metabolic dysregulation^19^. Metabolic dysregulation refers to disturbances or imbalances in the normal metabolic processes of an organism. These disruptions can occur in the metabolism of carbohydrates, fats, and proteins, leading to a cascade of clinical symptoms. For instance, disorders in carbohydrate metabolism can result in impaired fasting blood glucose levels or even lead to the development of type 2 diabetes^20^. Moreover, it is well-established that cognitive impairments can be caused by declining brain metabolism^21^. Strikingly, impaired glucose metabolism is one of the most frequently reported deficits in the AD brain given the widespread use of PET scanning in research^22,23^. Despite significant research into the metabolic disturbances associated with diseases like stroke and Alzheimer’s disease (AD), effectively intervening to address these risk factors remains challenging. One major obstacle is the complex metabolic heterogeneity and interplay between cells within the disease process. A systematic understanding of intra-tissue metabolic heterogeneity and cooperative mechanisms in stroke and AD is yet to be established^24^. Single-cell transcriptomic sequencing (scRNA-seq) has emerged as a powerful tool for investigating the intricate interplay between cell-specific transcriptional states and disease phenotypes^25–27^. Given the strong connection between transcriptomic and metabolomic profiles, scRNA-seq data enables a systematic assessment of the metabolic heterogeneity of cell subtypes and critical metabolic pathways during disease progression^28,29^. In this study, we comprehensively assessed metabolic shifts at the single-cell level across IS, HS and AD by merging single-cell datasets and utilizing a graphneural network-based method for evaluating metabolic fluxes (scFEA)^24^. The identification of metabolic process patterns that are shared by the three illnesses will assist in the understanding of the underlying mechanisms of neurodegenerative disorders.

## Results

### Data integration and profiling of single-cell data

**Fig. 1:**
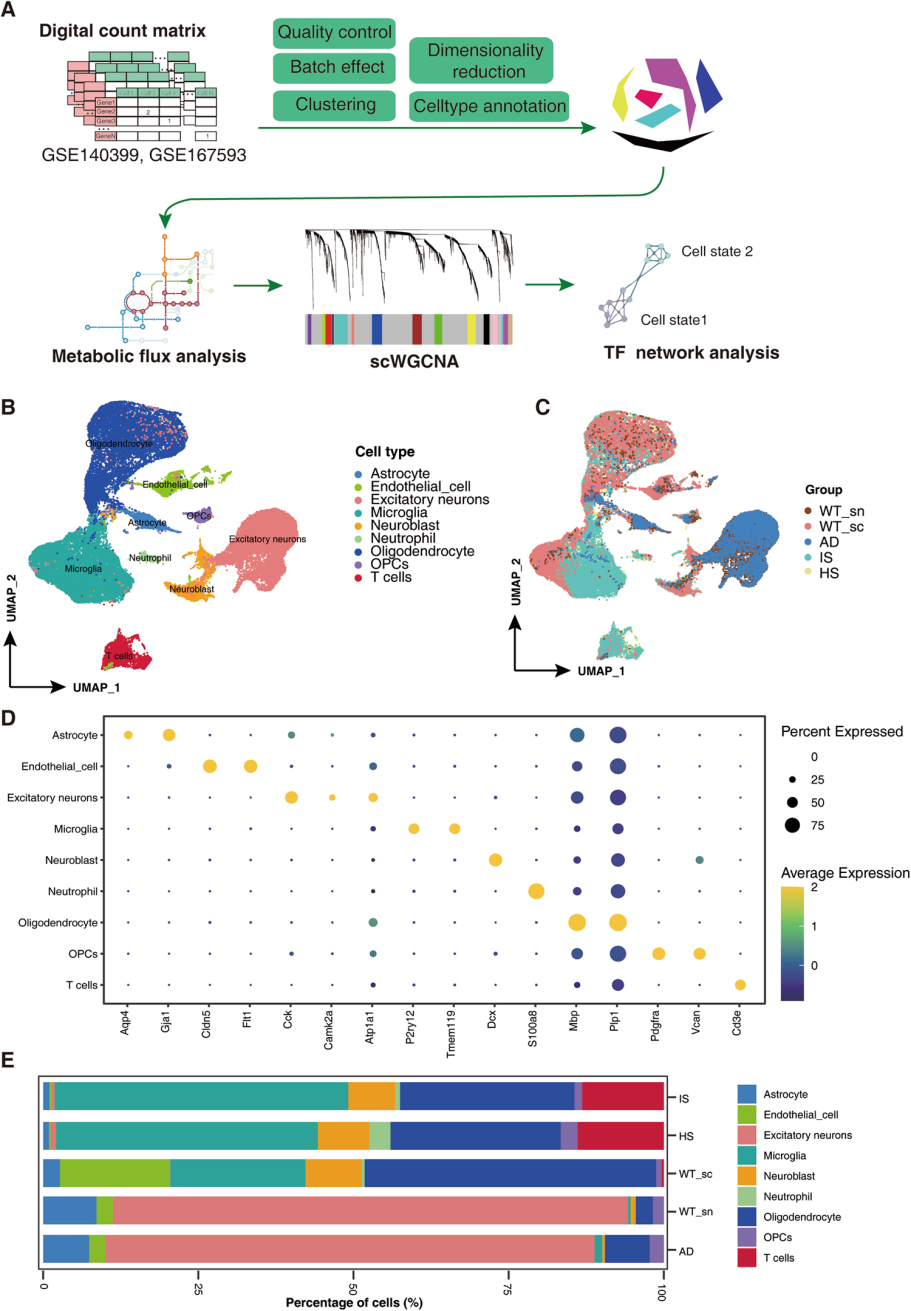
Single cell expression atlas of IS, HS and AD. (**A**) The overall analysis workflow of this study. (**B,C**) Uniform manifold approximation and projection (UMAP) plot for cell types and sample groups. WT_sc, Wild type with single cell sequencing. WT_sn, Wild type with single nucleus sequencing. (**D**) Dot plot displaying the average scRNA-seq expression of selected marker genes across all detected cell types (normalized to a scale of 0–2). E. Proportion of cells of each cell type (x axis) detected with different groups (y axis).

An overview of the data processing workflow is shown in Fig. 1A. In total, 51237 cortex cells from normal mice and three murine disease models of ischemic stroke, hemorrhagic stroke and Alzheimer’s disease were collected; Table 1 summarizes the sample information and cell numbers. After quality control and filtering, 49516 cells were utilized for further single-cell analysis. We then conducted dimensionality reduction and clustering analysis and identified nine cell types (Fig. 1B). The marker genes in Fig. 1D were chosen from published literature^30,31^. Compared to the WT_sc group, the proportions of endothelial cells were decreased in the HS and IS groups (Fig. 1C,E). This finding can be attributed to the severe disruption of vascular structure that occurs under conditions of ischemic stroke and hemorrhagic stroke^32,33^. Furthermore, compared to the WT_sc group, the HS and IS groups showed a notable increase in the proportion of T cells, suggesting the presence of evident infiltration of adaptive immune cells in the pathological conditions of ischemic stroke and hemorrhagic stroke. Additionally, the WT_sn group had a higher proportion of neurons than the WT_sc group, indicating that a single nucleus sequencing can capture more neurons.

**Table 1:** Basic characteristics of single-cell datasets.

Sample	GEO ID	Cell count before QC	Cell count after QC	Library Building Kit
WT_sc	GSM5111158	10636	10262	scRNA
WT_sn	GSM4160645	10548	10398	snRNA
AD	GSM4160643	4811	4767	snRNA
IS	GSM5111159	15136	14398	scRNA
HS	GSM5111160	10106	9691	scRNA

WT: wild type; AD: Alzheimer’s disease; IS: ischemic stroke; HS: hemorrhagic stroke; scRNA: single-cell RNA sequencing; snRNA: single-nucleus RNA sequencing; QC: quality control.

### Application of scFEA analysis revealed distinct metabolic states across different cell types

**Fig. 2:**
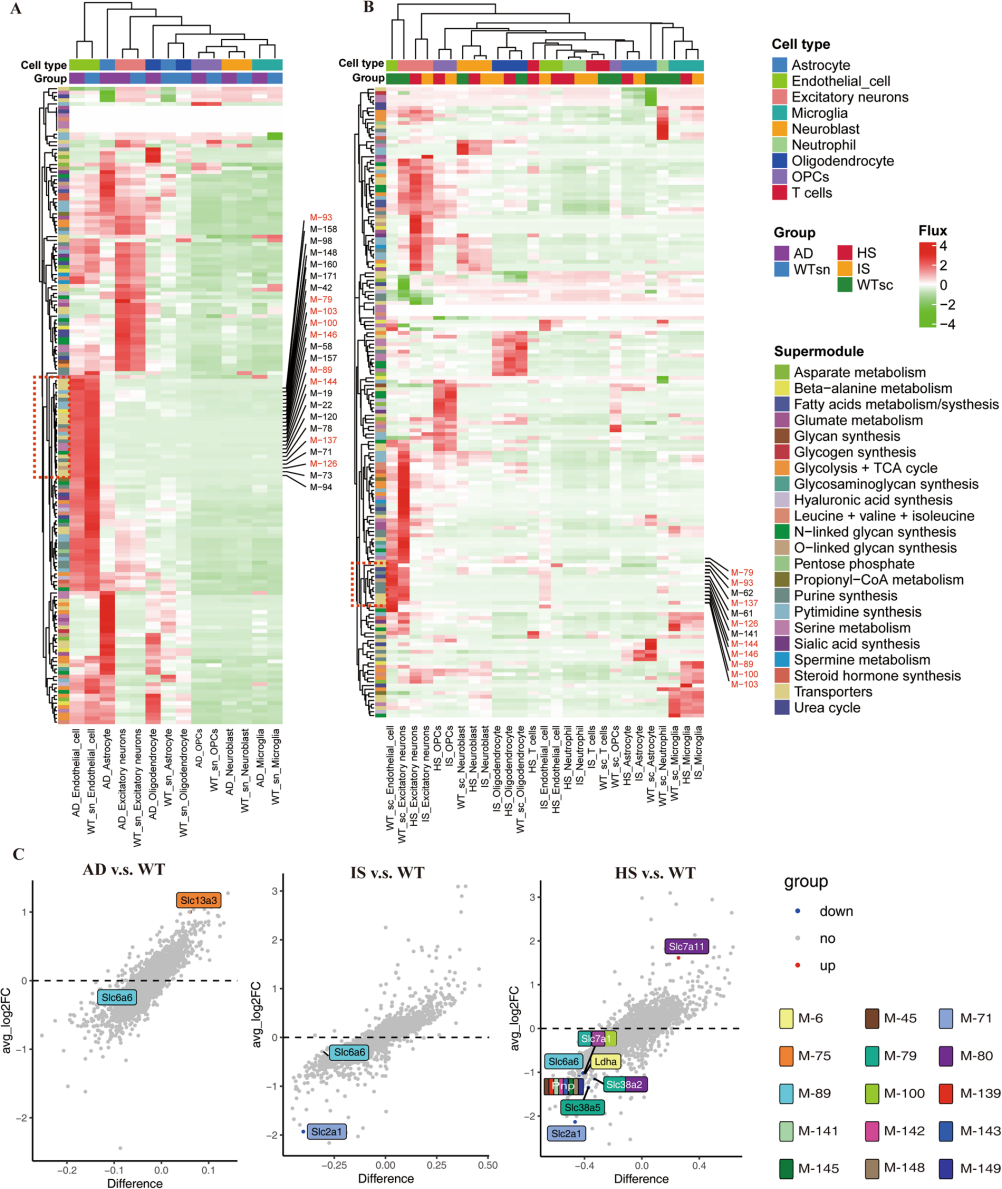
Single-cell metabolic flux mapping reveals the heterogeneity of metabolism between cell types and diseases. (**A,B**) Profile of the predicted fluxome of 168 metabolic modules. On the y-axis, each row displays the flux of a particular metabolic module for all the cells under various group cell type conditions (shown on the x-axis). The color scale bar represents the size and direction of metabolic fluxes. (**C**) Volcano map revealing differences in enzyme expression in endothelial cells in different diseases. The vertical axis provides average log2FC values, and the horizontal axis presents the difference between proportions. The color of each dot represents the direction of change. Enzymes that differ significantly are highlighted on the figure, and coloured annotations are added to the metabolic modules that they participate in.

The interaction and heterogeneity of cell metabolism are recognized to have a crucial role in the development of disease^34,35^, and discovering potential causes of neurodegenerative disease progression can be aided by the systematic investigation of metabolic heterogeneity and common characteristics across three disorders, IS, HS, and AD, which can result in neurodegenerative diseases. Therefore, we systematically evaluated 168 metabolic modules and 70 metabolites at the single-cell level using an integrated algorithm, called single-cell flux estimation analysis (scFEA)^24^. According to scFEA analysis, differences in cell types rather than disease situations were what primarily contributed to the heterogeneity of metabolic modules (Fig. 2A,B, Supplementary Table S1-2^36^). Although the fluxes of some metabolic modules of astrocytes and oligodendrocytes were significantly elevated in AD, there were no similar changes in IS and HS. Conversely, fluxes of some other metabolic modules of OPCs were significantly elevated in HS and IS but not in AD. This may be related to disease differences among AD and IS and HS.

Interestingly, the fluxes of several endothelial cell metabolic modules revealed a simultaneous decline in AD, IS, and HS, including M-79 (serine_in → serine), M-89 (B-alanine_in → B-alanine), M-93 (GABA_in → GABA), M-100 (arginine_in → arginine), M-103 (proline_in → proline), M-126 ((Gal)1 (GlcNAc)1 (Man)1 (Ser/Thr)1 → (Gal)1 (GlcNAc)1 (Man)1 (Neu5Ac)1 (Ser/Thr)1), M-137 (IMP → XMP), M-144 (hypoxanthine → xanthine) and M-146 (xanthine → uric acid), most of which belong to the transporter superfamily. Figure 2C shows the enzymes of the 168 metabolic modules with significantly altered expression levels in different disease models. The enzymes mediating M-79, M-89 and M-100 were significantly decreased in hemorrhagic stroke, further validating the results of metabolic flux. Although the expression of Slc6a6 catalysing M-89 was not significantly different between AD and IS, it showed a decreasing trend. Given that abnormalities in Slc6a6 can lead to metabolic syndrome^37^, this suggests that Slc6a6 may play a crucial role in neurodegenerative diseases. The above results collectively suggest that under the pathological conditions of stroke and AD, endothelial cells experience inhibition in transporter and amino acid transport pathways on their cell membranes. To provide more robust validation for these findings, we conducted additional analyses using gene set enrichment analysis algorithms on the GO and REACTOME databases (Fig. S1). The results of these analyses unveiled significant downregulation of the REACTOME_Slc_Mediated_Transmembrane_Transport pathway and amino acid transport-related pathways in the endothelial cells of both HS and IS groups. Moreover, the AD group displayed a similar downward trend in the GO_Regulation_Of_Amino_Acide_Transport pathway. These outcomes further corroborate the reliability of the insights gained from the scFEA analysis.

**Fig. 3:**
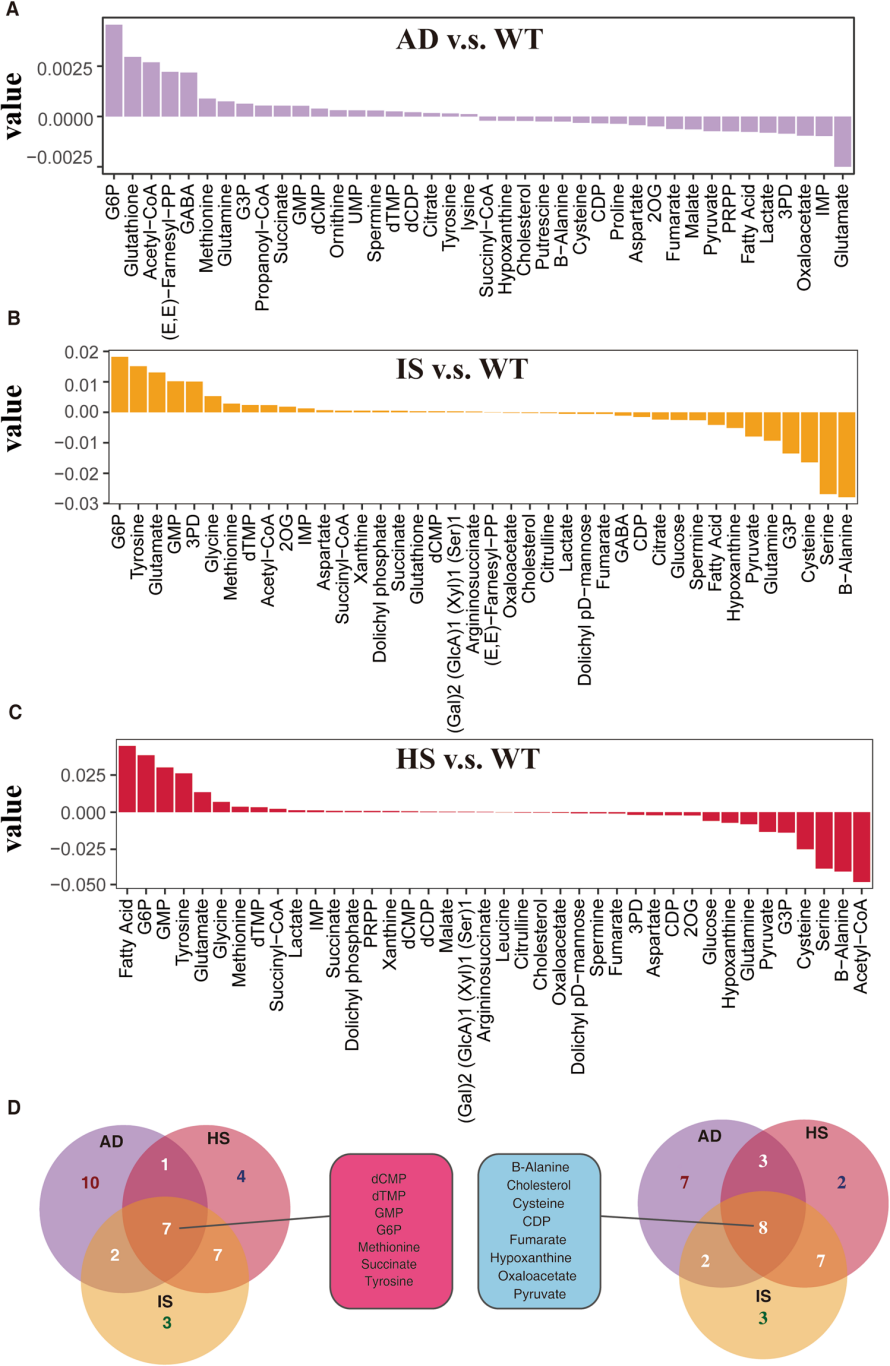
Endothelial cell metabolites accumulated and were depleted with great overlap in AD, IS and HS. (**A**–**C**) Top 20 metabolites predicted to accrue and deplete in the AD, IS, and HS groups when compared to the WT group. Metabolites are arranged on the x-axis in decreasing order of accumulation level. The metabolic stress level is represented on the y-axis, where a positive number indicates accumulation and a negative value indicates depletion. (**D**) Venn diagram showing the overlap of accumulated and depleted metabolites in the three diseases.

In addition, we also assessed the alterations in metabolite levels, and certain metabolites displayed a similar pattern in endothelial cells from AD, IS, and HS (Fig. 3, Supplementary Table S3–5). For instance, in all three groups, the product B-alanine of the M-89 module was reduced, and the levels of B-alanine have been demonstrated to have an inverse relationship with dementia^38^. Moreover, the concurrent decrease in pyruvate and fumarate, which are components of the tricarboxylic acid (TCA) cycle of glycolysis, aligns with previous research indicating the significance of glycolytic dysregulation in neurodegenerative diseases^39^. This, to some extent, validates the accuracy of the metabolic predictions made in this study. Given the consistency of endothelial cell metabolic dysregulation across the disease progression of HS, IS, and AD, and the fact that all three conditions lead to neurodegenerative outcomes^4,7^, the downregulation of endothelial cell metabolism may play a crucial role in neurodegeneration.

### Identification of perturbed coexpression module associated with altered endothelial cell metabolism in HS, IS, and AD

**Fig. 4:**
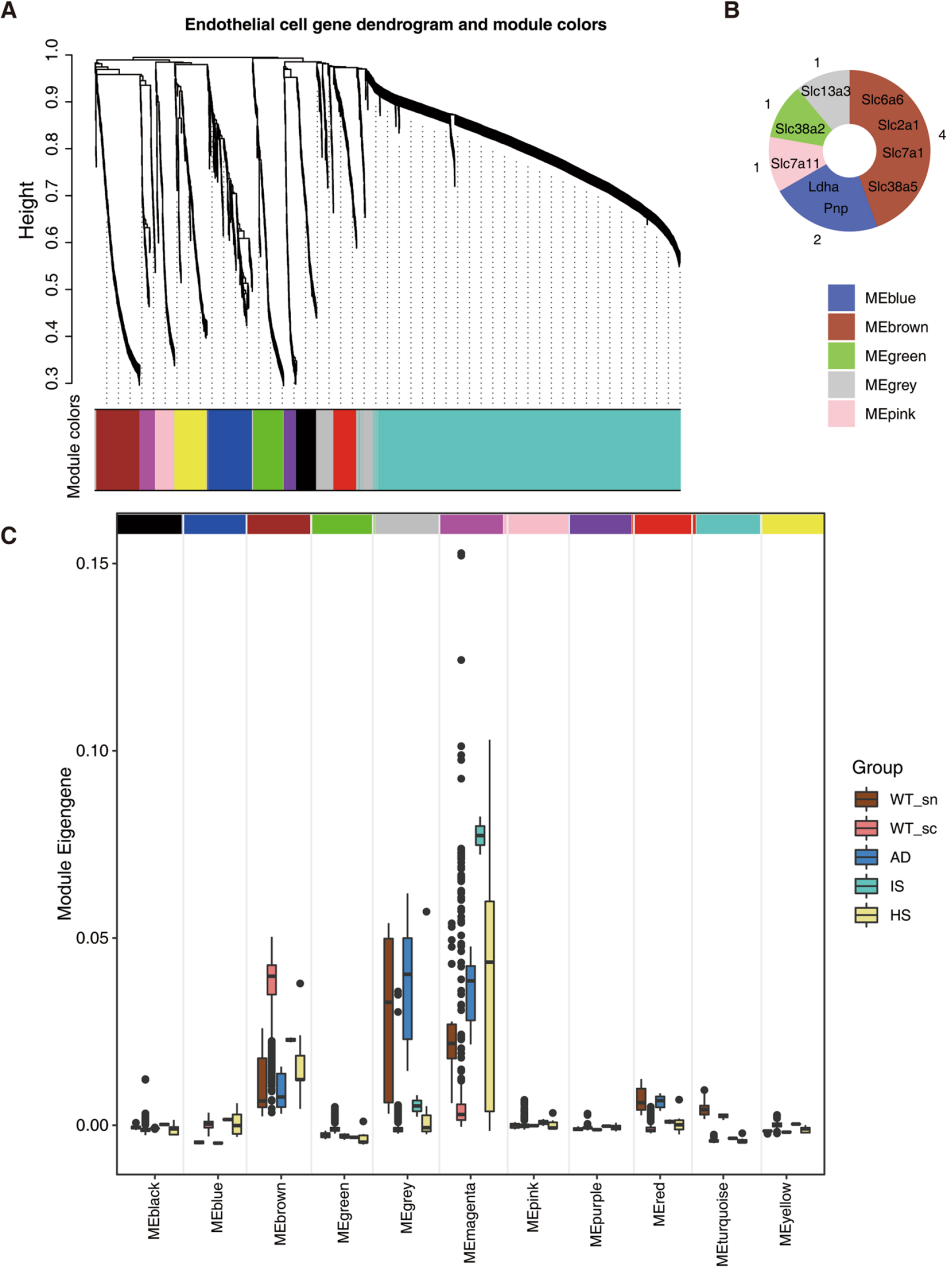
WGCNA identified a particular module associated with metabolic shift. (**A**) WGCNA modules and the clustering dendrogram. On the basis of the pattern of gene expression, the clustering diagram and 11 modules are shown. (**B**) Distribution of differential enzymes identified by the metabolic profile in the WGCNA module. (**C**) Box plot visualization of the eigengenes of various modules in different groups. The x-axis shows different gene coexpression modules, and the box colour represents the group. The y-axis shows the expression of the eigengene.

**Fig. 5:**
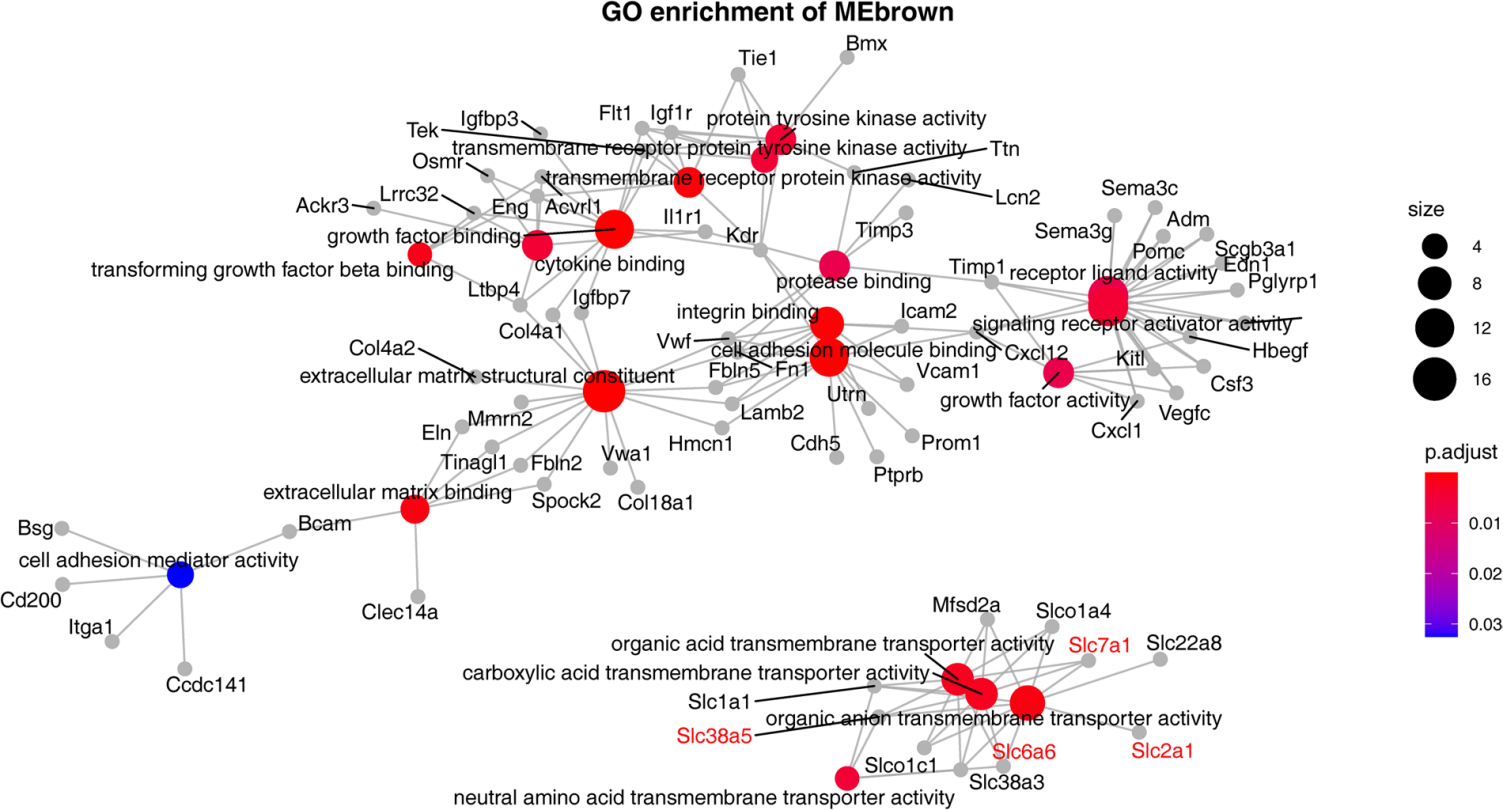
The specific module discovered by WGCNA was highly enriched in cell surface receptor- and ion channel-related pathways. Network of enriched pathways and genes. The colour of the dot represents p.adjust, and the size of the dot represents the number of genes enriched in the specific module of the pathway where the dot is located.

In our quest to uncover the underlying metabolic alterations in endothelial cells across HS, IS, and AD, we performed scWGCNA^40^ using 3000 highly variable genes obtained through the Findmarkers function from the Seurat Package^41^. This approach led to the identification of eleven distinct coexpression modules (Fig. 4A,B). The majority of the enzyme modules for the nine enzymes in Fig. 2D that had substantial changes belonged to the MEbrown module. Our subsequent comparison of the levels of various expression modules across different endothelial cell groups revealed that the expression levels of the MEbrown module in IS, HS, and AD were all lower than what in the WT group (Fig. 4C). To gain deeper insights into the biological implications of this coexpression module, we subjected its genes to GO enrichment analysis (Fig. 5). The results highlighted a prominent enrichment of genes associated with critical cellular processes, including signaling receptor activator activity, receptor ligand activity, and growth factor binding. Additionally, we observed an enrichment of genes involved in transmembrane transporter channel activity-related pathways, such as carboxylic acid transmembrane transporter activity and organic acid transmembrane transporter activity. This intriguing finding suggests that the metabolic shift observed in endothelial cells across these neurodegenerative conditions may be intricately linked to the dysregulation of specific pathways mediated by the MEbrown coexpression modules. The downregulation of these pathways in HS, IS, and AD could potentially play a pivotal role in the development and progression of neurodegenerative diseases.

### Transcriptional regulatory network analysis revealed key transcriptional regulators that cause metabolic shifts in endothelial cells

**Fig. 6:**
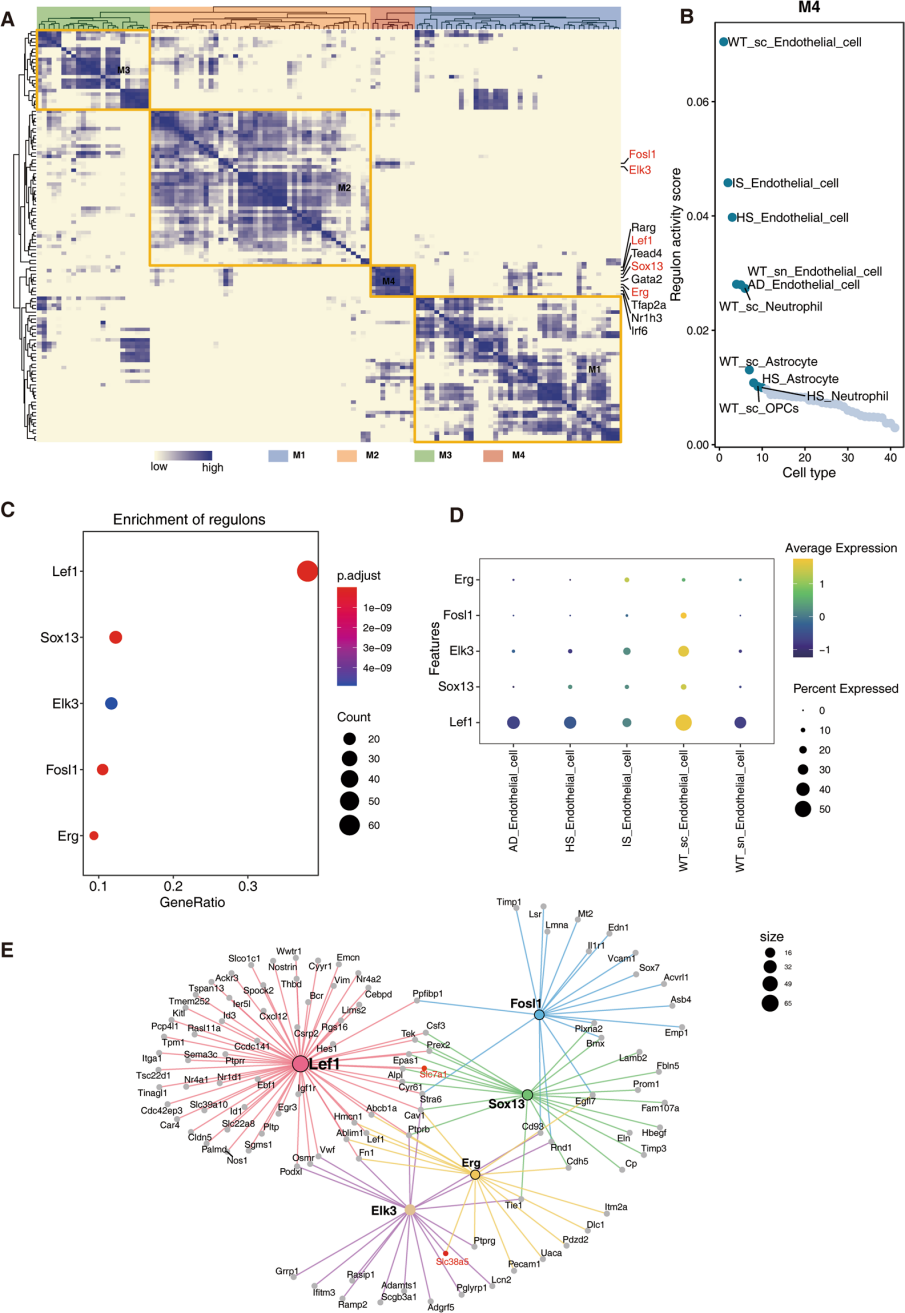
SCENIC analysis identified transcriptional regulatory networks that could lead to changes in metabolism. (**A**) Hierarchical clustering heatmap of the discovered regulons. Different modules are represented by various colors. (**B**) Scatter plot of M4 module transcriptional regulon activity scores ranked by different cell types. (**C**) Dot plot showing the top 5 regulons specifically enriched by genes in the MEbrown module identified by WGCNA. The color of the dot represents p.adjust. The size of the dot represents the count of genes enriched in regulons. (**D**) Dot plot displaying the average expression of identified transcriptional regulators across all detected cell types (normalized to a scale of 0–2). (**E**) Network of the top 5 regulons. Each line connects an upstream transcriptional regulator to a downstream target, and the colors of the lines correspond to different regulons.

Additional research into upstream transcriptional regulators that lead to altered metabolic changes may yield potential targets for therapeutic interventions to curtail the course of neurodegenerative disorders. To achieve this goal, we performed single-cell transcriptional regulatory factor analysis (SCENIC). Four modules comprising a total of 119 transcriptional regulators were found (Fig. 6A, Supplementary Table S6^36^). Figure 6B and Fig. S2 depict the sequencing of the activity score of these modules in various cells. Endothelial cells were found to have the most active form of M4, and IS, HS, and AD were all reduced relative to WT. To further clarify the transcription factors regulating the MEbrown module in Fig. 4, we examined the extent to which the genes of the MEbrown module were enriched in transcription factor targets using a hypergeometric test algorithm. The top 5 enriched transcriptional regulators were Lef1, Sox13, Elk3, Fosl1 and Erg (Fig. 6C). Among them, Lef1, Sox13 and Erg belong to M4 module, and Elk3 and Fosl1 are also related to transcriptional regulators in M4 module. Additionally, Fig. 6D verified the changes in these transcription factors based on their expression levels, further supporting their pivotal roles in the regulatory network. The transcriptional regulatory network in endothelial cells is demonstrated in Fig. 6E, where some targets of the network such as Slc7a1 and Slc38a5 are involved in the metabolism of transmembrane channels. Our findings elucidate the critical involvement of specific transcriptional regulators, particularly those within the M4 module, in orchestrating the metabolic shifts observed in endothelial cells.

## Discussion

In the current study, we integrated and constructed single-cell metabolic flux profiles of ischemic stroke, hemorrhagic stroke and Alzheimer’s disease for the first time. Our study revealed a simultaneous decline in specific metabolic modules in endothelial cells across ischemic stroke (IS), hemorrhagic stroke (HS), and Alzheimer’s disease (AD). The downregulation of B-alanine and intermediates of glycolysis and the tricarboxylic acid (TCA) cycle in these conditions suggests that dysregulation of transporter-related pathways may play a significant role in the onset and progression of neurodegeneration.

The finding that B-alanine levels were reduced across all three conditions is particularly intriguing, given its demonstrated inverse relationship with dementia^38^. B-alanine is a non-essential amino acid known to be involved in various physiological processes, including neurotransmission and the synthesis of carnosine, an antioxidant with neuroprotective properties^42,43^. Therefore, targeting B-alanine metabolism and its related pathways may represent a promising avenue for therapeutic intervention in neurodegenerative diseases.

Furthermore, a notable observation from our study is that the reduction in pyruvate and fumarate, crucial intermediates of glycolysis and the TCA cycle, was consistently observed specifically in endothelial cells across the three neurodegenerative conditions: IS, HS, and AD (Fig. 3). This intriguing finding highlights the unique role of endothelial cell metabolism in the pathogenesis of neurodegeneration. Endothelial cells form an essential component of the blood-brain barrier (BBB) and play a critical role in maintaining brain homeostasis^44^. Their metabolic state is tightly linked to their regulatory function in providing essential nutrients and energy substrates to neurons and glial cells^45^. Impaired endothelial cell metabolism can compromise BBB integrity, leading to increased permeability and subsequent infiltration of immune cells and inflammatory mediators into the brain, which can contribute to the progression of neurodegeneration^46^.

Glycolysis is not only a central pathway for energy production but also a critical source of precursors for biosynthetic processes essential for cell survival and function^47^. Previous studies have shown that metabolic dysregulation of glucose metabolism is present in both Alzheimer’s disease (AD) and stroke patients^48–51^. Our finding of reduced glycolysis specifically in endothelial cells in the context of IS, HS, and AD underscores the importance of studying cell-specific metabolic alterations to gain a comprehensive understanding of neurodegenerative disease mechanisms. Targeting endothelial cell metabolism, particularly glycolysis and related metabolic pathways, may hold promise as a potential therapeutic strategy to restore cellular energy balance, bolster BBB integrity, and mitigate neurodegeneration.

We also explored the upstream transcriptional regulators whose targets were significantly enriched in coexpression modules associated with metabolic shifts in endothelial cells (Figs. 4, 6), and by analysis of their gene expression levels we found that Lef1, Elk3 and Fosl1 were significantly decreased in stroke and AD. Lymphoid enhancer-binding factor 1 (Lef1) is a member of the Lef/Tcf transcriptional regulator family that mediates beta-catenin-dependent transcription in the Wnt-beta-catenin pathway and promotes endothelial cell proliferation and migration^52,53^. Studies have shown aged animals failed to initiate the expression of this EC migration pathway by day 14 post-stroke^54^. Additionally, Lef1 is involved in driving the expression of glucose-dependent insulinotropic peptide, which regulates glucose and energy homeostasis^55^. Therefore, Lef1 may play a crucial role in metabolic dysregulation in endothelial cells. Similarly, Elk3 and Fols1 have also been shown to be involved in endothelial adhesion and migration^56–58^. Moreover, the direct targets of the above transcription factors all involve enzymes such as Slc7a1 and Slc38a5 that underlie metabolic shifts in endothelial cells; therefore, the reduced transcriptional activity of Lef1, Elk3 and Fosl1 in endothelial cells may be the main cause of metabolic shifts. Intervention with these upstream regulators may be a potential approach to mitigate the process of neurodegeneration.

There are several unique aspects of this study. First, it systematically creates metabolic flow profiles at the single-cell levels for IS, HS, and AD. A focus on the same metabolic change during the disease process will assist in elucidating the potential metabolic pathways during neurodegeneration because neurodegeneration is implicated in all three disease pathologies. To identify potential targets for intervention to halt the course of neurodegeneration, this study also investigated the transcriptional regulatory networks that lead to metabolic alterations. However, this study still has several shortcomings. First, since all of the disease models in this study were based on mice, they do not accurately reflect the reality of the disease. Second, the transcriptional regulatory targets identified in this work were not further confirmed. Future research will broaden the types of diseases and construct more spectral single-cell metabolic profiles of neurodegenerative diseases and experimentally validate potential targets for intervention.

In conclusion, our investigation has provided novel insights into the shared metabolic shifts in endothelial cells across ischemic stroke, hemorrhagic stroke, and Alzheimer’s disease. Additionally, we have identified Lef1, Elk3, and Fosl1 as prospective targets for reversing the development of neurodegeneration. These findings hold promise for advancing our understanding of neurodegenerative disorders and may pave the way for future therapeutic strategies aimed at combating these debilitating conditions.

## Methods

### Data collection

The single-cell data of ischemic and hemorrhagic strokes^59^ was acquired from the Gene Expression Omnibus database (GEO; https://www.ncbi.nlm.nih.gov/geo/) via accession number GSE167593^60^. The single-cell transcriptome profiles of Alzheimer’s disease were retrieved using the accession numbers GSE140399 from GEO^61^. In these data, 5XFAD mice were used to simulate the disease state of AD^30^.

### Single cell data processing

After obtaining the raw single-cell count matrices, we merged and processed the data using the R package Seurat (version 4.0; https://satijalab.org/seurat/). To eliminate potentially low-quality or compromised cells, we filtered out those with UMI counts below 200 and a mitochondrial gene expression ratio exceeding 20%. Additionally, we removed genes expressed in fewer than three cells to address potential artifacts and reduce noise. The harmony package (https://github.com/immunogenomics/harmony) was utilized to remove batch variations between samples. Next, we conducted data normalization using standard procedures. The raw gene counts of each cell were adjusted by dividing them by the total number of reads in that particular cell, accounting for sequencing depth variations. Subsequently, a logarithmic transformation was applied to the normalized gene expression data to stabilize variance across the dataset and mitigate the impact of highly expressed genes. Next, we proceeded with dimensionality reduction, clustering, cell type annotation, and visualization steps to obtain unsupervised clustering of cells and their spatial distribution, following the guidelines provided on the Satija Lab website (https://satijalab.org/). The identification and annotation of clusters was performed using data that had already been published^30,31^.

### Construction of a single-cell metabolic flux profile

The acquisition of single-cell metabolic flux profiles mainly utilized a graph neural network algorithm called single-cell flux estimation analysis (scFEA)^24^. The 168 metabolic modules whose data were utilized in the research were directly downloaded from the algorithm’s official Github page (https://github.com/changwn/scFEA).

### Differential expression analysis and functional enrichment analysis

The genes that were upregulated in the AD, IS, and HS groups relative to the wild-type (WT) group across different cell types were determined using Seurat’s FindMarker function. Gene Ontology (GO) enrichment analysis and Gene Set Enrichment Analysis (GSEA) were carried out using the hypergeometric test and the clusterProfiler R package.

### Weighted gene coexpression network analysis (WGCNA)

Using the scWGCNA program^41^, we conducted WGCNA on endothelial cells. In this research, the top 3000 variable genes were taken into account. The signed adjacency matrix was generated for the purpose of identifying the gene module with SoftPower set to 10. Average hierarchical clustering served as the foundation for both genetree clustering and eigengene clustering.

### Network analysis for transcriptional regulators

Based on mm10 refseq-r80 500 bp up and 100 bp down tss.mc9nr.feather, single-cell regulatory network inference and clustering (SCENIC) analysis was carried out^62^. The SCENIC procedure was run with default settings, and the raw count matrix from all of the samples was utilized as input. The analysis was conducted in three steps. Prior to identifying the transcription factors (TFs) with direct targets (regulon), we calculated the coexpression modules and assessed the weight between the TFs and their target genes. AUCell was used to assess each regulon’s activity within each cell. The enricher function of the clusterprofiler package was used to run a hypergeometric test to identify regulons enriched in a certain module. For visualization, ggplot2 was used to calculate the average regulon activity scores of the regulon modules for each cell type and to create a rank plot of the regulons.

### Supplementary information

Supplementary Information

## Data Availability

In this study, publicly accessible datasets were analyzed. These links will take you to the information: https://www.ncbi.nlm.nih.gov/geo/query/acc.cgi?acc=GSE140399(ref. ^61^), https://www.ncbi.nlm.nih.gov/geo/query/acc.cgi?acc=GSE167593(ref. ^60^). We release our supplemental data at 10.5281/zenodo.8162047 (ref. ^36^).

## Code availability

The source codes used in this work are accessible on GitHub (https://github.com/GuangyuGuoer/scMF-across-AD-and-stroke).
