# Supplementary material for: Shared metabolic shifts in endothelial cells in stroke and Alzheimer’s disease revealed by integrated analysis
Source: Sci Data. 2023 Sep 29;10:666. doi: 10.1038/s41597-023-02512-5 (PMC10542331; doi:10.1038/s41597-023-02512-5)
Supplement: Supplementary file 1 — Supplementary Information [file 41597_2023_2512_MOESM1_ESM.pdf]

Supplementary Information

Shared metabolic shifts in endothelial cells in stroke and Alzheimer's disease revealed by integrated analysis

Guangyu Guo[1,2,3], Liyuan Fan[1,4], Yingxue Yan[3,4], Yunhao Xu[3,4], Zhifen Deng[3], Miaomiao Tian[3], Yaoqi Geng[3,5], Zongping Xia[1,2,3 *], Yuming Xu[1,2 *]

1. Department of Neurology, The First Affiliated Hospital of Zhengzhou University, Zhengzhou, Henan, China;

2. NHC Key Laboratory of Prevention and treatment of Cerebrovascular Diseases

3. Clinical Systems Biology Laboratories, the First Affiliated Hospital of Zhengzhou University, Zhengzhou, China

4. Academy of Medical Sciences of Zhengzhou University, Zhengzhou, China

5. Department of Endocrinology, The First Affiliated Hospital of Zhengzhou University

* These two authors are co-corresponding authors.

Table of Contents

Figure S1 GSEA Reveals Downregulation of Amino Acid Transport-Related Pathways in Endothelial Cells under Stroke and AD Disease Conditions

Figure S2 The regulon activity scores of different cell types among modules (M1, M2 and M3)

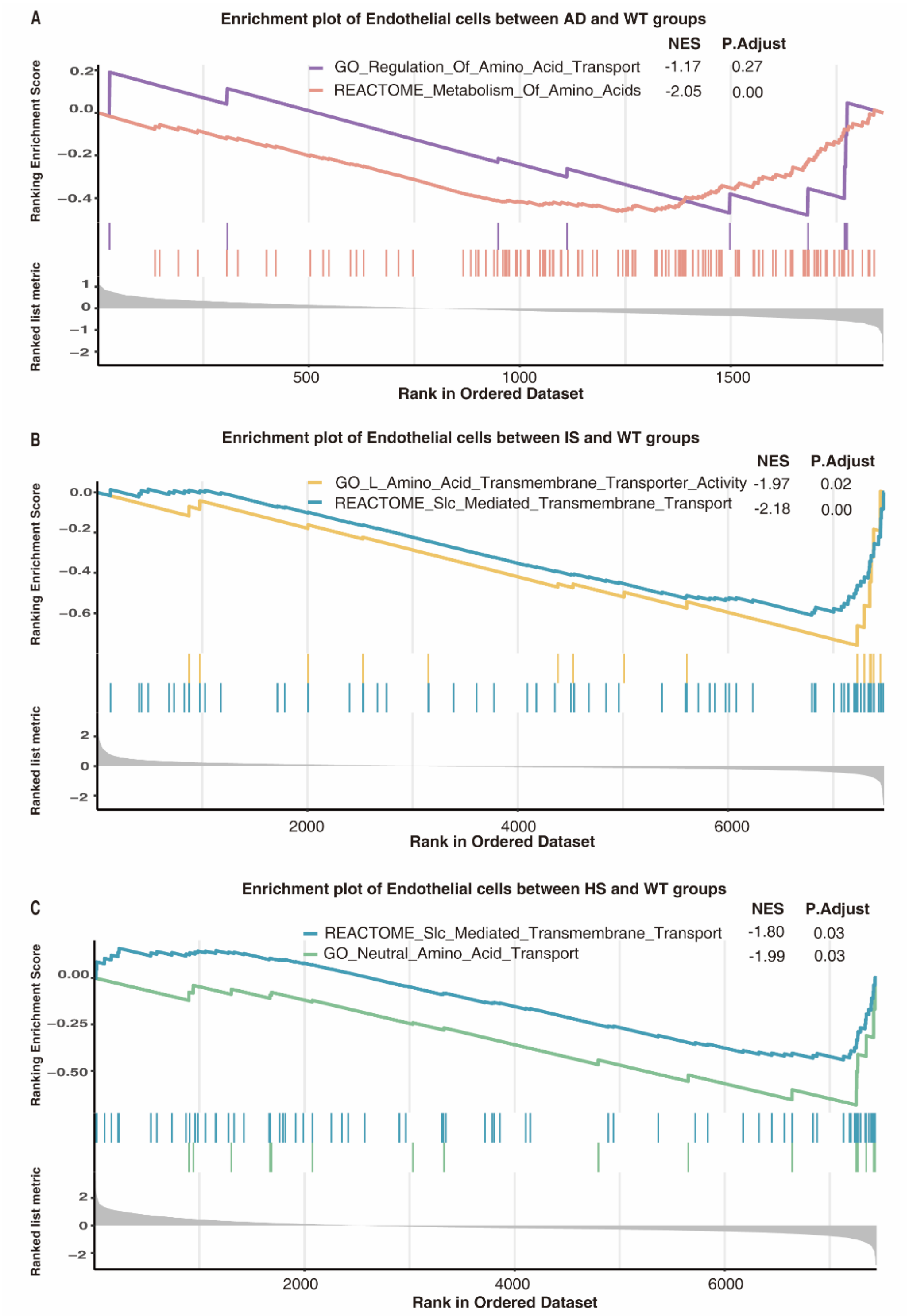

Figure S1. GSEA Reveals Downregulation of Amino Acid Transport-Related Pathways in Endothelial Cells under Stroke and AD Disease Conditions. Enrichment plot obtained from GSEA showing the dysregulated amino acid transport pathways in endothelial cells of AD (A), IS (B), and HS (C) groups. Each colored curve corresponds to a different pathway, with NES (Normalized Enrichment Score) indicating the extent of enrichment, and P.Adjust representing the Bonferroni-adjusted p-value.

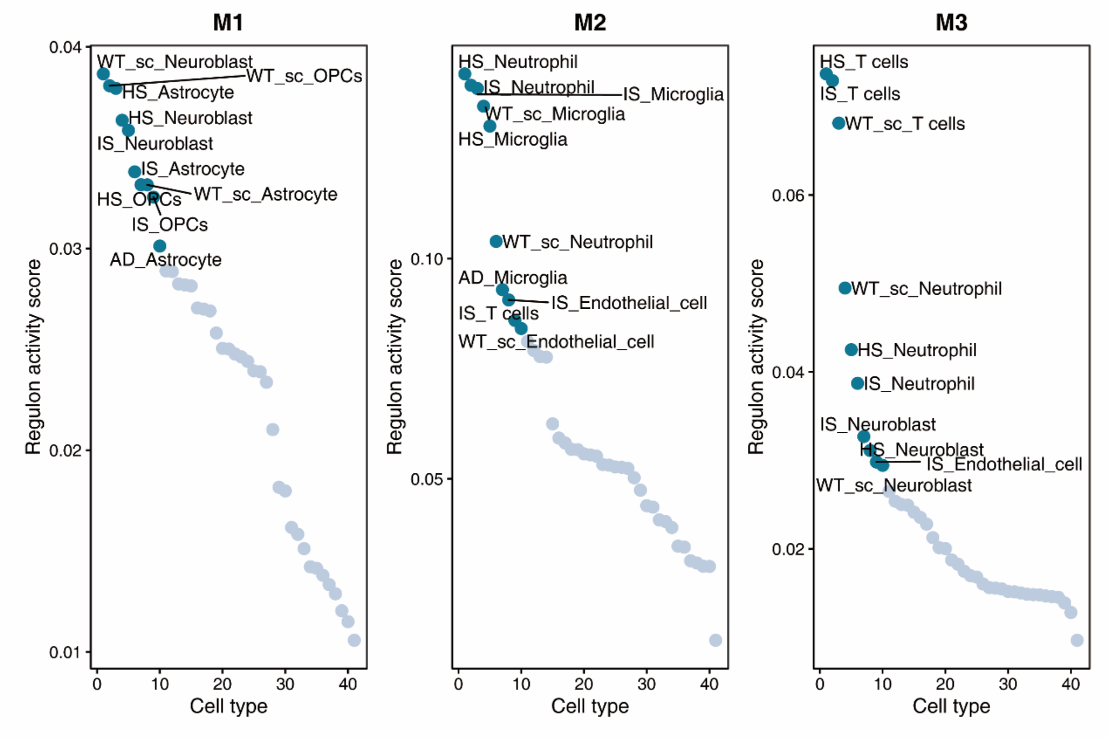

Figure S2. The regulon activity scores of different cell types among modules (M1, M2 and M3). Different cell types are shown on the x-axis, while the transcript activity score is shown on the y-axis.
